# Contrast-enhanced ultrasound for determining muscular perfusion after oral intake of L-citrulline, L-arginine, and galloylated epicatechines: A study protocol

**DOI:** 10.1097/MD.0000000000022318

**Published:** 2020-10-09

**Authors:** Julian Doll, Franziska Bürkle, Arndt Neide, Stefanos Tsitlakidis, Thomas Bruckner, Gerhard Schmidmaier, Christian Fischer

**Affiliations:** a Center for Orthopedics, Trauma Surgery and Spinal Cord Injury, Ultrasound Center, HTRG - Heidelberg Trauma Research Group, Heidelberg University Hospital; b Institute of Medical Biometry and Informatics, University of Heidelberg, Heidelberg, Germany.

**Keywords:** biceps muscle, contrast-enhanced ultrasound, green tea extract, L-arginine, L-citrulline malate, muscle perfusion, VASO6

## Abstract

**Introduction:**

The market for dietary supplements in the sports sector has been growing rapidly for several years, though there is still lacking evidence regarding their claimed benefits. One group is that of nitric oxide increasing supplements, so-called “NO-boosters,” which are claimed to improve the supply of oxygen and nutrients to the muscle by enhancing vasodilation.

The aim of this study was to investigate 3 of these supplements in healthy male athletes for their muscle perfusion-enhancing potential using contrast-enhanced ultrasound (CEUS).

**Methods:**

This placebo-controlled, double-blind, randomized cross-over trial will be carried out at the Center for Orthopedics, Trauma Surgery and Spinal Cord Injury of the University Hospital Heidelberg. Three commercial NO enhancing products including 300 mg of the specific green tea extract VASO6 and a combination of 8 g L-citrulline malate and 3 g L-arginine hydrochloride will be examined for their potential to increase muscular perfusion in 30-male athletes between 18 and 40 years and will be compared with a placebo. On each of the 3 appointments CEUS of the dominant biceps muscle will be performed at rest and after a standardized resistance training. Every athlete receives each of the 3 supplements once after a wash-out period of at least 1 week. Perfusion will be quantified via VueBox quantification software. The results of CEUS perfusion measurements will be compared intra- and interindividually and correlated with clinical parameters.

**Discussion:**

The results of this study may help to establish CEUS as a suitable imaging modality for the evaluation of potentially vasodilatory drugs in the field of sports. Other supplements could also be evaluated in this way to verify the content of their advertising claims.

**Trial registration:**

German Clinical Trials Register (DRKS), ID: DRKS00016972, registered on 25.03.2019.

## 1 Introduction

Recently, the supply and the use of dietary supplements increase tremendously at all levels of athletic sports. Unfortunately, these much-courted supplements are often used without a full evidence-based understanding of the potential benefits and risks associated with their use.

A vast number of supplements like proteins, vitamins, carbohydrates or plant extracts are distributed promising several ergogenic effects. Especially supplements aimed at increasing the production of nitric oxide (NO) in blood vessels (so called NO-boosters) make up one of the most popular supplement categories in the sport industry today.

In endothelial cells, the enzyme isoform endothelial NO synthase (eNOS) catalyzes the conversion of L-arginine into L-citrulline and NO.^[1]^ Among multiple effects on muscle physiology, NO acts as an endothelium-derived relaxing factor with vasodilator properties while elevating intracellular cyclic guanosine monophosphate (cGMP) levels in smooth muscle cells.^[1–3]^ Referring to this, products in this supplement category claim to improve the supply of oxygen and nutrients to the muscle via vasodilation and increased blood flow.

This supplement category includes, among others, L-citrulline, L-arginine, and epicatechin gallates.

L-arginine is considered a semi-essential or conditional proteinogenic amino acid,^[4]^ which is endogenously synthesized mainly in the kidney from L-citrulline.^[5]^ Furthermore, L-arginine is crucial for the normal function of the urea cycle, in which ammonia is detoxified and removed via metabolism into urea.^[6]^

The effect of orally administered L-arginine on L-arginine plasma levels is limited, owing to its liver and particularly intestinal first-pass metabolism.^[5,7,8]^ At the same time, oral intake of L-citrulline respectively its combination with L-arginine has been suggested to increase plasma L-arginine levels more efficiently than oral L-arginine alone, which was explained by inhibitory effects on catabolizing arginase enzymes as well as the fact that dietary L-citrulline is not metabolized by the liver and thus can serve as precursor of L-arginine in other tissues, for example, the kidney.^[8–11]^ Ultimately, this effect was associated with increased substrate availability for NOS.^[8]^

Epicatechin gallates (ECG) are different components of botanical green tea or grape seed extracts. Botanical extracts contain numbers of phytochemicals which may provide health benefits. These phytochemicals can be isolated via fractionation.^[12]^ Another product that is distributed in the category of NO-boosting supplements is “VASO6” (Serious Nutrition Solutions, 2965 Franklin Turnpike, Danville, VA). This green tea extract is advertised as consisting of specific catechins which have attracted great interest as vasodilating agents (product characteristics of VASO6 ^[13]^), based on previous studies on procyanidins derived from extracts of grape seeds (GSE).^[12,14]^ In 2002, single compounds of GSE fractions were bioassayed for endothelium-dependent relaxing (EDR) activity using the aortic ring model.^[14]^ As isolated procyanidins showed most potential with NOS-mediated EDR activity increasing with the degree of polymerization, epicatechin content, and with galloylation,^[14]^ specific procyanidin oligomers were proposed accordingly for the use in patients, especially the trimer-gallate epicatechin-(4–8)-epicatechin-(4–8)-epicatechin-gallate (C1-gallate).^[15]^

Previous studies have investigated the impact of these supplements advertised as “NO-boosters” on muscle blood flow or blood volume in resistance trained subjects with diverging results,^[16–19]^ making it challenging to draw a clear conclusion. Compared with the conventional examination tools that have been used in these studies such as strain-gauge plethysmography or Doppler-US, the increasingly applied technique of Contrast-Enhanced Ultrasound (CEUS) allows for a direct visualization of the microperfusion at higher resolution.^[20,21]^ The contrast agent SonoVue, which is commonly used for CEUS examinations, consists of microbubbles with a phospholipid shell and a sulfur hexafluoride core, an echogenic, poorly soluble gas.^[22]^ Due to their size (comparable to that of red blood cells),^[20]^ SonoVue microbubbles remain intravascular after injection and oscillate measurably upon collision with ultrasonic waves, generating contrast-specific signals for (subsequent) quantification.^[21,23]^

SonoVue is considered very safe with a low incidence of side effects.^[24]^ The microbubbles are extracted from the lungs within 10 to 15 minutes by exhaling and the phospholipid shell is metabolized by the liver.^[21]^ It does not interact with the kidneys. Thereby, it does not demand laboratory testing of renal function before administration and can be used in patients with renal dysfunction, when contrast agents for computed tomography (CT) or magnetic resonance imaging (MRI) are contraindicated.

Given the application of highly standardized examination algorithms, perfusion kinetics can thus be depicted in a reliable way.^[25]^ The muscle perfusion quantified by CEUS allows insights into muscle (patho-)physiology that cannot be assessed by conventional MRI protocols.^[26]^

In the past, CEUS has successfully been used to quantify skeletal muscle macro- and microperfusion and to assess the vitality of the skeletal muscle by means of the perfusion as a surrogate parameter in real time.^[26–29]^ However, to our knowledge this method has not yet been used to investigate the impact of pre-workout dietary supplements on muscular microperfusion. For this purpose, the following study will examine 3 different commonly used NO-boosting products via CEUS:

- 300 mg VASO6
- 8 g L-citrulline malate
- 3 g L-arginine hydrochloride

After oral intake, changes of muscular microperfusion linked to resistance training will be quantified and the effects of supplementation will be compared with one another and with placebo in 30 healthy athletes.

The study protocol for this study is described in the present manuscript.

## 2 Methods/design

### 2.1 Objectives

The primary objective is to generate hypotheses on the effects of selected supplement containing beverages on CEUS perfusion measurement in the resting and exercised biceps muscle compared with placebo. Therefore, the results of CEUS measurement will be compared intraindividually. In addition to that, the results of all participants will be sorted by beverage A, B, and C and tested for significance.

Moreover, several clinical parameters including body size, body weight, and age will be correlated with the results of CEUS analysis.

In future, CEUS could be an appropriate examination method to evaluate further potentially vasoactive supplements regarding their claimed impacts and benefits, especially in the sports sector. The current pilot study may thus contribute to initiate the implementation of CEUS in this field of research.

Except for a compensation of 100 Euros, the participants have no self-interest or any personal advantages due to the study participation.

### 2.2 Study design, registration, and ethics

The study protocol was approved by the local ethics committee (S-094/2019). Furthermore, it was registered at the German Clinical Trials Register (DRKS00016972). The study protocol will be conducted according to the Declaration of Helsinki.

This is a registered, randomized placebo-controlled double-blind cross-over single-center trial.

### 2.3 Inclusion and exclusion criteria

Young male athletes aged between 18 and 40 years with periodical sporting activity in popular or serious sport are eligible for inclusion after giving their written consent.

Exclusion criteria are recent myocardial infarction, cardiac or respiratory insufficiency, uncontrollable hypertension, severe respiratory disease (pulmonary arterial hypertension [PAH], severe acute respiratory syndrome [SARS]), marcumarization or bleeding disorders, liver, intestine or kidney diseases, and other known contraindications (e.g., allergies) to the contrast agent SonoVue as well as other similar contrast agents. Smokers in general will be also excluded. Additionally, persons who are not able to or do not agree to the informed consent will be excluded from the outset.

### 2.4 Enrollment of participants and study setting

The present study is a randomized placebo-controlled double-blind cross-over single-center trial.

Thirty healthy male athletes are to be enrolled in this study. Only male athletes will be included because of better comparability. The recruitment is realized by a written announcement on a board in university buildings or in sports facilities. Further information can be requested by phone or email.

Informed consent takes place in a face-to-face setting at the research site. A full verbal explanation of the study, a patient information sheet, and informed consent form will be provided in advance. Each athlete will be informed in detail about the scientific purpose and risks of the study, its voluntary nature, and the possibility to withdraw the agreement to participate in the trial at any time without giving any reasons. Athletes must be at least 18 years of age, without any exclusion criteria and provide their written agreement before any intervention. Moreover, the athlete's health status will be assessed by a medical examination prior to the first examination.

**Figure 1:**
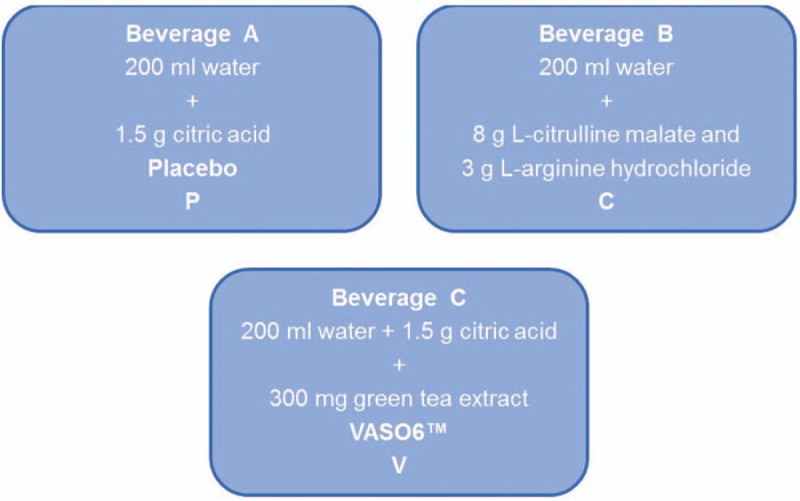
Overview of the different oral supplements. Beverage A: water with citric acid (placebo), Beverage B: combination of 8 g L-citrulline malate and 3 g L-arginine hydrochloride, Beverage C: 300 mg of a green tea extract with citric acid.

For every participant, the study includes 3 appointments for examination. In a randomized and double-blind manner, the athlete receives 1 out of 3 beverages per examination (Fig. 1), which always follows the same standardized procedure including CEUS perfusion measurement at rest and after exercising. The intervals between the study periods amount to at least 7 days.

**Figure 2:**
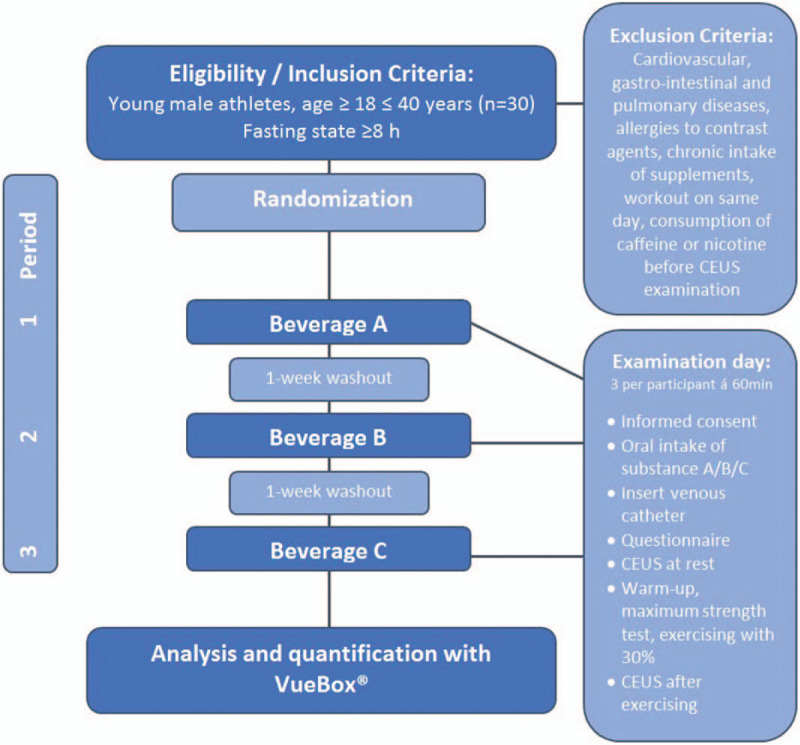
Flowchart of the trial.

The following procedure applies for each of the 3 study periods and is additionally illustrated in Fig. 2:

- Participants come after fasting overnight and at least for 8 hours to the appointment in our Center for Orthopedics, Trauma Surgery and Spinal Cord Injury.
- Oral intake of beverage A/B/C according to randomization.
- Inserting a 20-gauge cannula in the cubital vein.
- First CEUS examination (40 minutes after oral intake of the beverage) for evaluation of dominant biceps muscle perfusion at rest.
- Activation of the dominant biceps muscle according to a standardized exercise protocol (see below).
- Second CEUS examination (60 minutes after oral intake of the beverage, 60 seconds after exercise) for evaluation of biceps muscle perfusion after exercise.
- Removing of 20-gauge cannula, end of examination.

### 2.5 Exercise protocol

- Warm-up, maximum strength test of the dominant biceps muscle.
- Loading of the dumbbell corresponding to 30% of maximum strength.
- Concentric-eccentric exercise of the dominant biceps muscle in a standardized manner:
  ∘ 4 sets à 10 repetitions
  ∘ frequency of concentric/eccentric activation 1 Hz (accounting for 20 seconds load time per set)
  ∘ 60 seconds break between the sets.

The expenditure of time for every participant is about 60 minutes per appointment, travel time excluded.

In total, the time per participant for 3 examination appointments amounts 180 minutes, plus travel time.

### 2.6 Randomization

Every participating athlete will receive each of the 3 treatments once (3 study periods). For balancing purposes, they will be randomized in equal numbers to 1 of the 6 resulting treatment sequences (Table 1).^[30]^

**Table 1:**
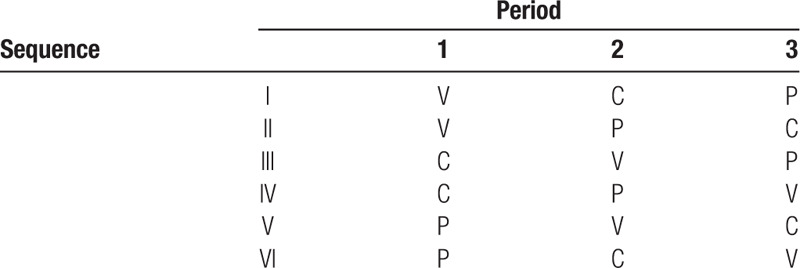
Randomization with 6 possible treatment sequences.

### 2.7 Study drugs and dosing

As we intend to investigate the perfusion-related effects of pre-workout supplements in a realistic setting, products that may be used in an athlete's everyday routine will be administered instead of pharmaceutical grade substances:

- 300 mg VASO6
  In the current study we examine the vasodilating potential of this specific green tea extract in a common dosage of 300 mg. This dose was considered to be safe and well tolerated in healthy subjects.^[31]^
- combination of 8 g L-citrulline malate and 3 g L-arginine hydrochloride
  We use the dietary amino acids to be examined in the common forms of L-citrulline malate and L-arginine hydrochloride.^[11,32]^ Former studies described short-term administration of L-citrulline dosages up to 15 g as well-tolerated without adverse effects.^[33]^ Doses over 15 g led to saturation effects during the increase of arginine plasma concentration (which was referred to renal arginine synthesis by the authors), thus lower dosages were recommended for clinical practice.^[33]^ Regarding the L-arginine dosing it has been reported that single doses of 3 to 6 g rarely caused, if any, gastrointestinal side effects such as nausea or diarrhea.^[34]^ Based on the favorable effect of combined L-arginine and L-citrulline supplementation as described above and in synopsis with common dosage recommendations of market-leading supplements, we therefore choose a combination of 8 g L-citrulline malate and 3 g L-arginine hydrochloride.

Each substance is dissolved in 200 mL water. Placebo treatment only contains 200 mL of water. For the purpose of blinding, the green tea extract and placebo treatments are each combined with 1.5 g citric acid to imitate the natural sour taste of the beverage that contains L-citrulline combined with L-arginine. Accordingly, 3 beverages will be administered with 2 of them containing supplement ingredients and 1 placebo (hereinafter defined as beverage A, B, or C as illustrated in Fig. 1). Every beverage is odorless. To cover up the brownish color of VASO6, black cups will be applied.

### 2.8 Instructions

Subjects will be instructed to maintain their weekly training routine throughout the study periods. At the first appointment, they will be asked about their diet on the preceding evening, which should be replicated before the second and third appointment.

During the week prior to the first study period and for the time of participation, subjects have to refrain from dietary intake of L-citrulline, L-arginine, or VASO6 beyond the trial. Training is permitted up to 24 hours prior to the examinations. The intake of caffeine is permitted up to 12 hours prior to the examinations.

### 2.9 CEUS examination

All CEUS examinations will be performed at the local university ultrasound center by the same experienced orthopedic and trauma consultant with DEGUM level III (German Society for Ultrasound in Medicine) qualification.

The CEUS video clips will be postprocessed and analyzed by an experienced orthopedic and trauma resident.

We will use the same, highly standardized examination protocol as reported previously.^[35]^

For examination of the biceps muscle, subjects will be positioned in a supine position, with their dominant arm abducted 70° in full supination on a positioning pillow. A 20-gauge cannula will be inserted in the contralateral cubital vein.

An ACUSON S2000 ultrasound device (Siemens Healthineers, Erlangen, Germany) will be used for all sonographic evaluations. A linear probe (9L4 probe, 4–9 MHz) will be positioned at the transition between the middle and distal third of an imaginary axis between the anterior axillary line and the medial epicondyle of the humerus, perpendicular to the course of the muscle fibers. The resulting cross-section comprises the humeral shaft, the brachial artery as well as the fascia separating the biceps and brachialis muscle bellies as standardized landmarks. Having identified the standardized plane in conventional B-mode, a live dual-view B-mode image in the Siemens-specific contrast Cadence mode will be enabled with gain set at 3 db and a mechanical index (MI) at 0.08. A 2.4 mL bolus of the contrast agent SonoVue will be injected through a 20-gauge cannula into a peripheral vein and flushed with 10 mL of 0.9% saline solution (NaCl). The described settings are in accordance with the recommendations of the EFSUMB^[36]^ and will be applied for each CEUS examination. A 70 seconds video clip with a frame rate of 5 Hz will be digitally recorded, starting with injection. In order to reduce artifacts, subjects are not allowed to move during the examination. At the end of the second CEUS examination, the cannula will be removed after the exclusion of any adverse events.

### 2.10 Quantification

The wash-in and wash-out of the contrast agent in the biceps muscle will be analyzed with the dedicated, commercially available quantification software VueBox (Bracco Imaging, Milan, Italy). The region of interest (ROI) will be positioned into the muscle tissue of the biceps muscle without any fasciae or large arteries to avoid distorting signals. The brachialis muscle will be selected by the same criteria as reference following the recommendations of Bracco Imaging and Tang et al.^[37]^ Based on a selected ROI within the muscle tissue, the resulting time–intensity curve provides the following perfusion-related parameters:

- Peak enhancement (a.u.): Maximum signal intensity of the enhancement curve,
- Wash-in area under the curve (a.u.): Definite integral of the signal intensity against time until peak enhancement,
- Rise time (s): Wash-in duration of the contrast agent,
- Time to peak (s): Duration from SonoVue application to peak enhancement,
- Wash-in rate (a.u.): Maximum slope of the signal enhancement curve,
- Wash-in perfusion index (a.u.): Ratio of wash-in area under the curve to rise time
- Wash-out area under the curve (WoAUC [a.u.], i.e., the definite integral of the signal intensity against time starting at peak enhancement),
- Wash-in wash-out area under the curve (WiWoAUC [a.u.], i.e., the sum of WiAUC and WoAUC).

Due to the pilot study character, it remains to be clarified which are the most suitable parameters to depict potential effects.

In addition to CEUS, several clinical parameters including body size, body weight, and age will be recorded.

### 2.11 Adverse events and risks for the participants

Pain, bleeding, hematoma (“blue spot”) or extravasation because of cannula (mal-) positioning are possible. Infections or injury to nerve tissue at the injection site are very rare. Furthermore, vasovagal reactions such as nausea, discomfort, dizziness, hypotension, sensation of warmth, or syncopation may occur. Previous reports on the applicability of SonoVue demonstrated a complication rate as low as 0.0086%.^[38]^

There are no known adverse events of the oral supplements in the dosage used in this study. Previous studies consider this dosages to be safe for healthy individuals and without adverse events.^[31,33,34]^ It has to be considered that travel expenses have to be paid by oneself.

### 2.12 Criteria that lead to termination of study

Every participant is able to withdraw their consent to participate in the trial at any time without giving reasons. Thereby the recorded study data may be destroyed immediately upon request or with the consent of the participant, can still be included in the evaluation.

If initial data indicates either impossible realization due to technical difficulties or an increased risk for the participants that is potentially harmful, the study will be terminated immediately.

### 2.13 Statistical analyses

The empirical distribution of continuous data and scores will be reported with means, standard deviation, median, minimum, maximum values, and with absolute and relative frequencies for categoric data. The statistical evaluation will be carried out with methods of variance analysis taking into account the crossover design.

The analyses and illustrations will be carried out by use of Excel for Windows (Microsoft EXCEL 2019, Redmond, WA), SPSS version 25.0 for 135 Windows (IBM Corp., Armonk, New York, NY), and GraphPad Prism version 6.00 for Windows (GraphPad Software, San Diego, CA). A *P*-value of ≤.05 will be used to indicate statistical significance. Due to the design of the study as exploratory data analysis, the calculated *P*-values have only descriptive value.

The details of the statistical analysis will be established in a statistical analysis plan, which will be completed before the closing of the database (before the end of data collection).

### 2.14 Sample size

Formal sample size determination is not possible as potential effects are not yet known. For the time being, the intended sample size is 30.

## 3 Discussion

The objective of this placebo-controlled, randomized cross-over study is to assess the muscle perfusion increasing potential of 3 different commercial NO enhancing supplements in 30 healthy male athlete's biceps muscle at rest and after exercising.

Previous studies have investigated the impact of specific procyanidins, L-citrulline, and L-arginine on nitric oxide production, vasodilating effects or exercise performance. While some of them were only performed in vitro or in animal models,^[9,14]^ others showed diverging results.^[16–18,32,39,40]^

For example, Gonzales et al^[16]^ measured artery blood flow with doppler ultrasound after L-citrulline supplementation during a standardized exercise and could show that L-citrulline has a modest effect of improving muscle blood flow.

Alvares et al^[18]^ monitored muscle oxygenation and blood volume with near-infrared spectroscopy after supplementation of L-arginine and exercising. They found out that acute L-arginine supplementation increases muscle blood volume during recovery from sets of resistance exercise with no increase in strength performance.

In 2016, Alsop and Hauton^[39]^ examined in their study the effect of nitrate and citrulline supplementation on cardiac electrical activity and blood flow via infrared (IR)-plethysmography. They could demonstrate, that nitrate and citrulline supplementation decreased vascular tone.

However, Bloomer et al^[40]^ compared Glycine Propionyl-L-Carnitine (GlycoCarn) and 3 different “nitric oxide stimulating” pre-workout nutritional supplements, whose constituents include among others L-arginine and L-citrulline, via measurement of skeletal muscle oxygen saturation (StO2), blood nitrate/nitrite (NOx), lactate (HLa), malondialdehyde (MDA), and exercise performance in men. They reported that none of the products tested in their study resulted in any statistically different effects. The tested products were ineffective in terms of increasing blood flow and improving acute upper body exercise performance.

Furthermore, Tang et al^[17]^ investigated in 2011 the ergogenic potential of L-arginine on NO synthesis, muscle blood flow, and skeletal muscle protein synthesis (MPS) in healthy young men. They concluded, that L-arginine does not increase NO, muscle protein synthesis, or muscle blood flow.

The variety of positive as well as negative results that were observed in terms of vasodilating effects may in part be explained by the considerable methodological differences^[11]^ including the applied methods of perfusion measurement and the derived parameters.

To our knowledge, this is the first study quantifying muscle perfusion changes after oral intake of these different supplements via CEUS in a human collective. Due to its high resolution limit, CEUS increases the sensitivity of conventional imaging methods^[20]^ and may thus be a promising way to detect even small changes of muscular microperfusion following oral supplementation.

In this study a combination of orally administered L-citrulline and L-arginine were selected due to the fact, that some studies described a more effective and rapid increase of plasma L-arginine and augmented NO-dependent response.^[8,9,41]^ Furthermore, the addition of malate to dietary L-citrulline was chosen in this study, since previous studies observed higher levels of NO metabolites with the addition of malate.^[11,42]^ Dosages of 8 g L-citrulline malate and 3 g L-arginine hydrochloride were used in this study, because previous studies consider these dosages to be safe for healthy individuals and without adverse events.^[31,33,34]^ It was observed in literature, that single doses of >15 g of L-arginine cause gastrointestinal side effects (i.e., diarrhea and vomiting). There is also evidence in literature that plasma citrulline and arginine levels reached a concentration maximum (*T*_max_) 0.7 to 1 hour after administration. Afterwards, they decreased to approximately baseline levels within 5 to 8 hours. Urinary excretion measured over 8 hours following administration was minimal.^[6,33]^

However, there exist few human studies about the tolerability of green tea extracts. Chow et al^[31,43]^ asserted that a dosage between 300 and 800 mg is safe for healthy individuals and causes no adverse events. According to the recommendation of the manufacturer of VASO6 and in accordance with the studies of Chow et al^[31,43]^ a common dosage of 300 mg was administered.

## 4 Article summary section

### 4.1 Strengths and limitations of this study

Nevertheless, we measure neither pharmacokinetic parameters (such as plasma arginine levels or NO metabolites) nor potential effects on exercise performance. Therefore, potential changes of microcirculation cannot be related to increased NO production as it is advertised by the vendors. Moreover, the implied association with better exercise performance would remain to be elucidated.

The selection of ROIs may be considered as further limitation, as they are to be positioned manually to exclude interfering vessels and fasciae. However, as biceps muscle perfusion quantification will be conducted under strictly standardized conditions, the acquisition of reliable measurements is feasible as mentioned above.^[25]^

The primary strength of this study is its placebo-controlled, randomized cross-over, and well-established design of high value.

Moreover, CEUS is a well-entrenched and experienced examination tool at our ultrasound center and will be performed by the same experienced orthopedic and trauma consultant with DEGUM level III (German Society for Ultrasound in Medicine) qualification. CEUS evaluates tissue microcirculation and appears to be a promising, non-invasive technique in the assessment of muscle perfusion.^[27–29]^ It completely avoids radiation and is a widely available, time- and cost-efficient method that permits the evaluation of the dynamic process of contrast enhancement within tissues in real-time. The ultrasound contrast agent SonoVue used for CEUS has been shown to have low complication rates when compared with CT and MRI contrast agents. It is neither nephrotoxic nor hepatotoxic and blood testing is not necessary prior to examination.^[44]^ Compared with other methods, CEUS has some advantages: due to its higher resolution limit at capillary level, it increases the sensitivity of conventional doppler ultrasound and can demonstrate flow velocity in real time. CEUS has fewer contraindications than dynamic MRI and is well tolerated.^[35,45,46]^

Sometimes the probe position has to be rotated or tilted to get the best imaging conditions.

The proposed study should help to reveal the vasodilating potential of selected NO-boosting products via CEUS by means of muscle microperfusion. Additionally, the current study might contribute to an establishment of CEUS as an appropriate examination tool for the evaluation of vasodilating agents in future.

## 5 Trial status

The RCT is ongoing (study protocol version 1.0 27.02.2019, S-094/2019), patient recruitment and examination began in October 2019. No follow-up is planned. Data analysis will only be performed after complete examinations and study end is expected to be completed end of October 2020. Thereafter, the final results will be published.

## 6 Additional file

**Figure 3:**
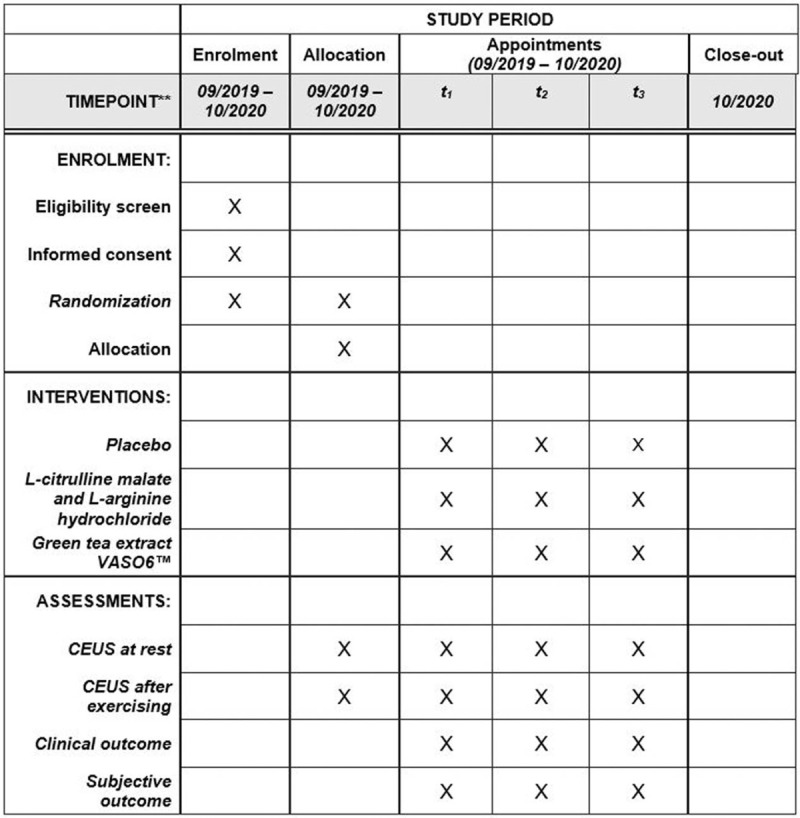
Study process schedule (according to the standard protocol items: recommendations for Interventional Trials [SPIRIT] guidelines).

Additional file 1: Standard Protocol Items: Recommendations for Interventional Trials (SPIRIT) 2013 Checklist: recommended items to address in a clinical trial protocol and related documents. (JPEG 108 kb). (Fig. 3 and Additional file 1).^[47,48]^

## Acknowledgments

The authors acknowledge financial support by the Baden-Württemberg Ministry of Science, Research and the Arts and by Ruprecht-Karls-Universität Heidelberg.

## Author contributions

**Conceptualization:** Julian Doll, Franziska Bürkle, Arndt Neide, Thomas Bruckner, Christian Fischer.

**Data curation:** Franziska Bürkle, Arndt Neide, Stefanos Tsitlakidis, Thomas Bruckner.

**Formal analysis:** Julian Doll, Franziska Bürkle, Arndt Neide, Stefanos Tsitlakidis, Thomas Bruckner.

**Investigation:** Arndt Neide, Stefanos Tsitlakidis.

**Methodology:** Thomas Bruckner.

**Project administration:** Julian Doll.

**Resources:** Christian Fischer.

**Software:** Thomas Bruckner.

**Supervision:** Stefanos Tsitlakidis, Gerhard Schmidmaier, Christian Fischer.

**Validation:** Thomas Bruckner, Gerhard Schmidmaier, Christian Fischer.

**Visualization:** Franziska Bürkle.

**Writing – original draft:** Julian Doll, Franziska Bürkle.

**Writing – review & editing:** Julian Doll, Gerhard Schmidmaier, Christian Fischer.

## Abbreviations

Abbreviations: a.u. = arbitrary units, CEUS = contrast-enhanced ultrasound, cGMP = cyclic guanosine monophosphate, CT = computed tomography, DEGUM = Deutsche Gesellschaft für Ultraschall in der Medizin, ECG = epicatechin gallates, EDR = endothelium-dependent relaxing, eNOS = endothelial NO synthase, MRI = magnetic resonance imaging, NO = nitric oxide, PE = peak enhancement, ROI = region of interest, RT = rise time, StO2 = oxygen saturation, TTP = time to peak, WiAUC = wash-in area under the curve, WiPI = wash-in perfusion index, WiR = wash-in rate, WiWoAUC = wash-in wash-out area under the curve, WoAUC = wash-out area under the curve.
